# Machine learning predicts lipid emulsion stability in parenteral nutrition using multi-laboratory literature data

**DOI:** 10.3389/fnut.2025.1668464

**Published:** 2025-11-17

**Authors:** Shang Yong-guang, Wang Xue-lian, Cheng Yong, Qin Wang-jun, Li Peng-mei, Zhang Lei

**Affiliations:** 1 Department of Pharmacy, China-Japan Friendship Hospital, Beijing, China; 2 School of Information, Beijing University of Chemical Technology, Beijing, China

**Keywords:** lipid emulsion stability, parenteral nutrition, XGBoost, SHAP interpretation, machine learning

## Abstract

**Objective:**

Physical instability of lipid in parenteral nutrition (PN) poses significant clinical safety risks. As lipid stability is influenced by multiple complex factors and remains incompletely characterized, this study aimed to quantify the relative importance of stability determinants and to develop a machine learning (ML) model for predicting stability in individualized PN prescriptions.

**Methods:**

A retrospective meta-analysis integrated experimental data from multi-laboratory studies. The ML framework employed transfer learning for cross-laboratory data harmonization and Synthetic Minority Over-sampling Technique (SMOTE) for class imbalance mitigation. Model performance was evaluated using the area under the receiver operating characteristic curve (AUC-ROC) and accuracy.

**Results:**

The datasets comprised 17 stability-related features (electrolytes, macronutrients, and storage conditions) extracted from 1,518 samples representing 872 unique PN formulations across 19 studies (2000 and 2024). The XGBoost model achieved exceptional predictive performance (accuracy: 98.2%, AUC 0.968). SHAP-based feature importance analysis identified the concentrations of Amino and phosphate, storage time and lipid composition as key stability determinants.

**Conclusion:**

This study establishes the first interpretable ML framework for predicting lipid emulsions stability in PN, resolving cross-laboratory data heterogeneity. We have provided a high-accuracy prediction tool for assessing lipid emulsion stability in PN, while the methodology demonstrates generalizability for stability studies of complex drugs and nutrients formulations.

## Introduction

Parenteral nutrition (PN) is liquid nutrition that is delivered directly to the bloodstream of patients unable to absorb adequate nutrients through the digestive system. PN simultaneously supplies macronutrients (amino acids, dextrose, and lipids), which constitute the caloric and protein supply, and micronutrients (vitamins and trace elements), which complement the diet. As one of the most complex preparations in hospital, PN carries inherent risks of physicochemical interactions among its components. These interactions can compromise emulsion stability, potentially leading to patient safety hazards.

Commercially intravenous lipid emulsion is thermodynamically unstable heterogeneous dispersions two-phase system. Oil droplets, primarily composed of triglycerides, are stabilized by a surfactant layer of phospholipids derived from egg lecithin. The phosphatidic acid outside the lipid droplets is completely ionized to form as zeta potential between −40 and −50 mV, which realizes mutual electrostatic repulsion, thereby preventing aggregation and maintaining a uniform distribution of lipid droplets. The addition of cations (e.g., Na^+^, K^+^, Ca^2+^, Mg^2+^) can neutralize this negative surface charge, reducing the zeta potential, lead to droplet aggregation and subsequent phase separation within PN. The critical aggregation number (CAN) is a parameter that tries to predict the amount of cationic charge that would disturb lipid emulsion. pH significantly influences zeta potential and stability. A pH range of 6.0–9.0 (1) is optimal for lipid emulsion stability. Consequently, low-pH additives like dextrose solutions can compromise stability (2), while amino acid solutions can exert a stabilizing effect by modulating the PN pH (2). Lipid emulsions become unstable at pH values below 5.0 (2). The American Society for Parenteral and Enteral Nutrition (ASPEN) stated in 2014 that PN containing >4% amino acids, >10% dextrose, and >2% lipid emulsion remained stable at room temperature for up to 30 h or refrigerated (5 °C) for up to 9 days followed by 24 h at room temperature (2), However, the 2024 ASPEN update provides only select concentration examples without comprehensive stability criteria (3). Similarly, other guidelines list validated PN formulations (4), but generally lack specific recommendations on macronutrient and electrolyte concentration limits essential for predicting physicochemical stability.

In summary, lipid emulsion stability in PN is influenced by a multitude of interacting factors, including electrolyte concentrations, macronutrient composition, pH, storage temperature, and duration. Furthermore, the inherent variability of individualized PN prescriptions means that existing stability studies are often limited to specific formulations. Consequently, a generalizable method for evaluating and predicting lipid emulsion stability across diverse clinical PN remains lacking in practice. To address this gap, the present study conducted a systematic overview of current research on lipid emulsion stability in PN. We extracted key features from these studies, detailing the analytical methodologies employed, the stability variables measured, and the principal factors investigated. Ultimately, this work aims to establish a clinically applicable prediction model for lipid emulsion stability in PN utilizing Machine Learning (ML).

## Materials and methods

### Study design

An ML-powered meta-analysis framework incorporating transfer learning techniques was developed to integrate cross-laboratory evidence. Studies investigating lipid emulsion stability in PN were systematically identified, screened, and subjected to original data. This process established a multidimensional ML database. Domain adaptation techniques were applied to address heterogeneous data distributions across laboratories. Subsequently, ML algorithms were employed to construct a lipid emulsion stability prediction model, with performance validated through cross-validation.

### Search strategy

A systematic literature search was conducted across PubMed, Embase, and Web of Science to identify relevant studies on PN stability. The search was restricted to English-language publications from 2000 to 2024. The following search syntax was employed: (“parenteral nutrition” OR “total parenteral nutrition” OR “total nutrition admixture” OR “all in one nutrition” OR “lipid emulsion”) AND (“stability/instability” OR “compatibility/incompatibility” OR “safety/unsafety”).

### Inclusion and exclusion criteria

Studies were included if they met the following criteria:

1. Experimental investigations of PN stability relevant to clinical practice.
2. Lipid emulsion stability had been analyzed at least one of Lipid droplet evaluation criteria following USP Chapter 729 <Globule Size Distribution in Lipid Injections>
  - PFAT5 (The percentage of weighted volume of fat residing in droplet of diameter >5 microns).
  - MDD (Mean droplet diameter).
3. Utilization of validated analytical methods.
  - Dynamic Light Scattering (DLS)/Photon Correlation Spectroscopy (PCS) or Laser Diffraction (LD) for MDD.
  - Light Extinction/Single Particle Optical Sensing (LE/SPOS) for PFAT5.

Studies were excluded if they:

1. Assessed only non-physical stability aspects (e.g., chemical degradation, precipitation).
2. Evaluated formulations containing non-standard compounding agents.
3. Analyzed lipid emulsion stability outside PN.
4. Focused solely on drug-PN compatibility.
5. Were conference abstracts, reviews, book chapters, or lacked accessible full text/raw data.

**Figure 1:**
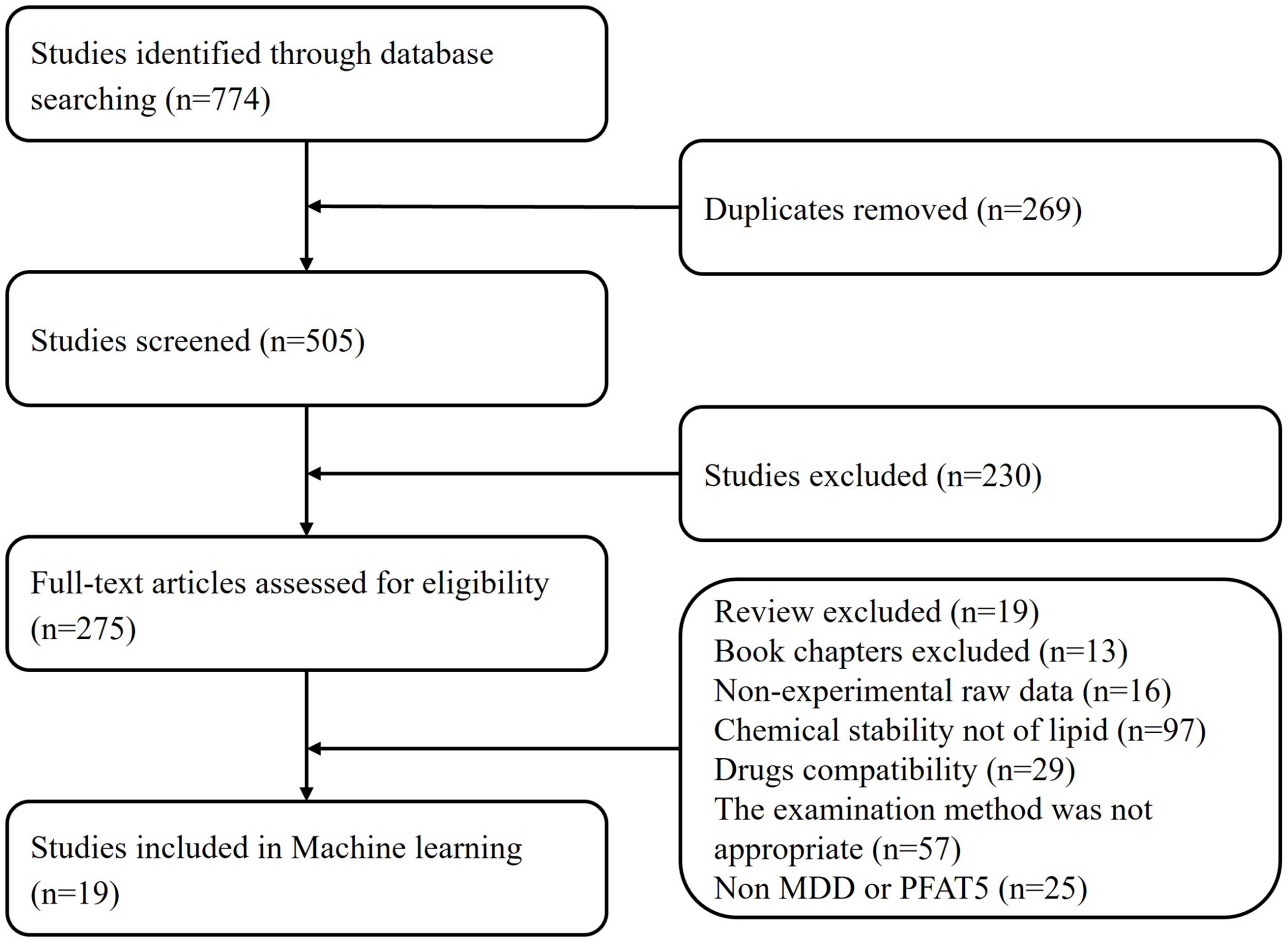
Studies inclusions and exclusions flowchart.

Flow diagram shown in Figure 1, summarizes the search strategy following PRISMA (5).

### Feature extraction

The features were extracted from the experimental studies reported in the literature including Environmental variables, PN composition characteristics and stability assessment results. Two researchers independently extracted the features data of experimental studies based on the following features definitions and formed the original dataset after cross-verification.

1. Environmental variables included:
  - Study year: In which year the study was conducted.
  - Temperature storage: Preparation and storage temperature.
  - Temperature test: The temperature during the stability analysis.
  - Times storage: The storage time of preparation.
  - Times test: The time for conducting the stability analysis.
2. PN composition characteristics:
  - Lipid composition: Long-Chain Triglycerides-LCTs, Medium Chain Triglycerides-MCTs or MCT/LCT blends in different proportions.
  - Addition of micronutrients (within or without) included: Fat-soluble vitamins, Water-soluble Vitamins, Trace elements, Heparin.
  - Concentration of electrolyte (mmol/L) included: Potassium; Sodium; Calcium; Magnesium; Phosphate; Chloride.
  - CAN: The critical aggregation number is a predictive parameter for cationic charge induced destabilization (*CAN = a + 64b + 729c*). a, b and c are the sum of the concentrations (mmol/l) of mono-, di- and trivalent cations.
  - Concentrations of Macronutrient (g/100 mL) included: Amino, Dextrose, Lipid.
3. Results of lipid emulsion stability assessment: The methods that were used for determination the stability of lipid emulsion was following the United States Pharmacopeia (USP), lipid globule size distribution be controlled within specified limits:
  - MDD: Instability threshold is MDD ≥ 0.5 μm (analytical methods: DLS/PCS or LD).
  - PFAT5: Instability threshold is PFAT5 ≥ 0.05% PFAT5 < 0.05% (analytical methods: LE/SPOS).

### Data preprocessing

1. Missing value handling: An automated protocol classified missingness as: Low (<5%), Moderate (5%–15%), High (15%–30%), and Extreme (>30%). Missing data mechanisms were evaluated using Little’s Missing Completely at Random (MCAR) test (*α* = 0.05). For variables with 5%–20% missingness, Multiple Imputation by Chained Equations (MICE) was implemented (10 iterations generating 5 imputed datasets).
2. Outlier detection & processing: A tripartite detection framework was employed: Modified *Z*-score (threshold: |Z| > 3.5); Isolation Forest (contamination = 0.05, n_estimators = 200, max_samples = 256, random_tate = 42); Local Outlier Factor (n_neighbors = 20, contamination = 0.05). A specific constraints rule based on the formulation of PN solution was established, and the violation of the rule was considered as abnormal: CAN >150, Amino acids (>1%), Glucose (>2%), Lipid emulsion (>1%), MDD > 50 nm, PFAT5 < 0.001% or >4%.
3. Dataset partitioning and leakage prevention: A strict random split was applied to partition the dataset: Training set *n* = 1,062 (70%), and Test set *n* = 456 (30%). This approach ensured clear separation, effectively preventing leakage of information. Parameters for all preprocessing steps (including SMOTE resampling, missing value imputation and feature scaling) were all determined before random split.
4. Multicollinearity handing: We calculated the Variance Inflation Factor (VIF) for all variables. We employed a stepwise approach: first removing variables with VIF > 10, for any remaining variable pairs with a correlation coefficient >0.8, retaining the variable deemed to have greater physical significance based on domain knowledge.

### Statistical analysis

Data were expressed as frequencies (percentages) for categorical variables and as Mean ± SD or median (IQR) based on the test of normality for continuous variables. The Kolmogorov–Smirnov test was applied to test normality. Differences between stability and instability groups were analyzed using Student’s *t*-test for continuous variables and Fisher’s exact test for categorical variables. A correlation heatmap was used to test potential multicollinearity between features. Statistical analyses and modeling were conducted using IBM SPSS Statistics (v25.0) and scikit-learn (v1.1) within Python 3.7 (Python Software Foundation; accessed January 1, 2025).

### Model development

1. Algorithmic diversity: Tree-based models (random forest, decision tree, XGBoost, gradient boosting), Non-tree models (logistic regression, support vector machine, K-Nearest neighbors, Naïve Bayes) and Ensemble/Neural (Multilayer Perceptron and AdaBoost).
2. Class imbalance mitigation: To address significant class imbalance (13.1% unstable emulsions), systematic evaluation of hybrid resampling techniques was implemented including: SMOTE, ADASYN, random under-sampling. Domain-informed optimization of sampling ratios enhanced minority-class (unstable emulsions) recognition performance across validated configurations.
3. Hyperparameter optimization: Model hyperparameters were systematically optimized using Bayesian methods to ensure robustness and stability.
4. Evaluation protocol: Model performance was comprehensively assessed using five core metrics: accuracy, precision, recall, F1-score, AUC-ROC. To ensure clinical reliability and enable transparent assessment of misclassification risks, particular emphasis was placed on a detailed confusion matrix analysis and class-specific performance metrics—including precision, recall, and F1-score—for both stable and unstable lipid emulsion categories, thereby enabling multidimensional validation of the model’s predictive capability.
5. Ensemble strategy: Final model outputs were weighted soft-voting aggregation of probabilistic predictions to enhance cross-laboratory generalization capability.

### Model interpretation

We employed SHAP values to quantify feature importance and interpret model predictions. Grounded in cooperative game theory’s Shapley values, SHAP provides consistent, theoretically-grounded attribution of feature contributions to individual predictions. Predictions. Model-specific implementations were utilized: Trees SHAP for tree models and Kernels HAP for non-tree models. Additivity was verified (tolerance <0.01), with ensemble SHAP values computed as weighted averages of base models. Global importance was ranked by mean absolute SHAP values, identifying top features.

## Results

### Data characteristics

The final analytical dataset comprised 1,518 PN samples representing 872 unique clinical prescriptions, extracted from 19 studies published between 2000 and 2024. These studies involve six different types of emulsions (three kinds of LCTs, two kinds of MCT /LCTs and one kind of SMOF lipid), storage temperature from 4 to 37 °C and storage time from 0 h to 14 days, the concentration of monovalent cation (Na^+^/K^+^) from 0 to 230 mmol/L, divalent cations (Ca^2+^/Mg^2+^) from 0 to 92.5 mmol/L. The concentration of macronutrient (mean ± SD): Amino (3.4 ± 2.0 g/100 mL), Dextrose (11.2 ± 6.2 g/100 mL), Lipid (2.8 ± 1.0 g/100 mL), Complete study characteristics are detailed in Table 1.

**Table 1:** Characteristics of studies included.

Reference	Year	Type of emulsion	Storage temperature and time	Analytical method	Droplet size measured	Monovalent cations mmol/L	Divalent cations mmol/L	Amino g/100 dL	Dextrose g/100 dL	Lipid g/100 dL	Main results
Driscoll et al. (14)	2000	100% Soybean oil 50% Soybean oil/50% MCT	4 °C/4 days RT/30 h	DLS SPOS	MDD PFAT5	70–140	15–30	6	16	2.3	50% Soybean oil/50% MCT controlled within USP limits
Driscoll et al. (15)	2001	50% Soybean oil/50% Safflower oil 100% Soybean oil 50% Soybean oil/50% MCT 80% Olive oil/20% Soybean oil	RT/30 h	LE SPOS	PFAT5	44.7	3.01	2.3	2.3	5	50% Soybean oil/50% Safflower oil 24 h and 100% Soybean oil 30 h PFAT5 > 0.05
Driscoll et al. (16)	2003	50% Soybean oil/50% Safflower oil 50%Soybean oil/50% MCT 50% MCT/ 40% Soybean oil/10% Fish oil	RT/48 h	LE SPOS	PFAT5	33–90	8.4–17.5	2.0–3.0	18.0–24.0	2.0–3.0	50% Soybean oil/50% Safflower oil 6 h PFAT5 > 0.05, 24 h exceeds 0.4%
Driscoll et al. (9)	2006	50% Soybean oil/50% MCT	6 °C/4 days 25 °C/30 h	LE SPOS	PFAT5	90–230	10–20	3.5–7.0	5.0–15.0	2.0–4.0	50% Soybean oil/50% MCT controlled within USP limits
Driscoll et al. (10)	2006	50% Soybean oil/50% MCT	RT/30 h	DLS SPOS	MDD PFAT5	102.4–189.8	12.8–23.8	7.1–7.7	19.6–21.3	2.5–2.7	Controlled within USP limits
Gonyon et al. (17)	2008	80% Olive oil/20% Soybean oil	5 °C/7 days RT/4 days	LD SPOS	MDD PFAT5	58	4.2	4.4	22.2	2.2	MDD < 0.5, PFAT5 < 0.01, and decreased with time
Skouroliakou et al. (18)	2008	100% Soybean oil	4–25 °C/7 days RT/48 h	LD	MDD	200	24.1	1.95	10.6	2.2	MDD controlled within USP limits
Chaieb et al. (19)	2008	80% Olive oil/ 20% Soybean oil 100% Soybean oil	37 °C/24 h	DLS	MDD	27–45	17.6–33.6	0.5–3.1	4.3–12.6	2	MDD controlled within USP limits
Driscoll et al. (20)	2010	50% Soybean oil/50% MCT	25 °C/30 h	LE SPOS	MDD PFAT5	29	16.5	1–4	5–10	2.0–4.0	Concentration of amino acid had a great influence on the stability
Skouroliakou et al. (21)	2012	30% Soybean oil/30% MCT/25% Olive oil/15% Fish oil	4–25 °C/24–48 h	DLS	MDD	31.1	13.5	1.1	8.9	1.5	MDD controlled within USP limits
Lobo et al. (6)	2012	50% Soybean oil/50% MCT	4–37 °C/7 days	DLS LO	MDD PFAT5	207	12.8	3	8	3	Trace elements have less effect than vitamins, and less effect than both add
Athanasiou et al. (22)	2013	30% Soybean oil/30% MCT/25% Olive oil/15% Fish oil 80% Olive oil/ 20% Soybean oil	4–25 °C/24 h	DLS	MDD	26.5–30.5	2.4–14	2.3–4.0	10.6–12.6	1.6–2.0	MDD controlled within USP limits
Cloet et al. (23)	2017	30% Soybean oil/ 30% MCT/25% Olive oil/15% Fish oil	2–8 °C/8 days RT/24 h	DLS	MDD	195	23	2.0	15.4	0.8	MDD controlled within USP limits
Sayed et al. (24)	2020	30% Soybean oil/30% MCT/25% Olive oil/15% Fish oil	25 °C/24 h	DLS	MDD	23–50	5.9–12.5	1–5.8	4.3–9.1	0.5–2.0	MDD controlled within USP limits
Zhao et al. (25)	2021	50% Soybean oil/50% MCT	RT/72 h	SPOS	MDD PFAT5	300.0	10.0	3.0	7.0	2.0	PFAT5 < 0.05, 24 h One of the manufacturers MDD > 0.5um
Gostynska et al. (26)	2021	50% MCT/40% Soybean oil/10% Fish oil 80% Olive oil/ 20% Soybean oil 100% Soybean oil 30% Soybean oil/ 30% MCT/ 25% Olive oil/ 15% Fish oil	2–8 °C/7 days	DLS	MDD	7.7–213.1	3.0–29.1	1.7–2.1	12.3–15	3.4–4.2	PN admixtures to remain stable for seven days within the specified limits
Gao et al. (27)	2021	50% Soybean oil/50% MCT	25 °C/24 h	SPOS	PFAT5	0–139	0–3.4	3.35–4.5	5–10	2.4	The addition of divalent ions upper 2.7 mmoL/L was unstable
Otero-Millán et al. (28)	2024	50% Soybean oil/50% MCT	4 °C-RT/14 days	DLS	MDD	100–180	30–92.5	2.5	7.5	0.12–2	Lipid emulsion concentration less than 0.25%, or amino acid concentration less than 2.5% RT 1–3 days MDD greater than 0.5
Otero-Millán et al. (29)	2024	50% MCT/40% Soybean oil/10% Fish oil	4 °C-RT/7 days	DLS	MDD	30–130	11.2–35	2.0–3.3	7.1–13.5	0.7–4.0	MDD controlled within USP limits

LD, laser diffraction; PCS, photon correlation spectroscopy; DLS, dynamic light scattering; LO, light obscuration; LE/SPOS, light extinction single-particle optical size; MCT, medium-chain triglyceride; RT, room temperature.

### Correlation between features and lipid emulsion stability in PN

Correlational analyses between extracted features and emulsion stability are presented in the correlation heatmap (Supplementary Figure S1). MDD analysis performed on 1,058 bags (69.7%), with instability (MDD ≥ 0.5 μm) detected in 32 samples (3.0% 32/1058). PFAT5 were determined 971 samples (64.0%), instability (PFAT5 ≥ 0.05%) observed in 173 samples (17.8% 173/971). Overall instability defined as violation of either USP threshold (MDD ≥ 0.5 μm or PFAT5 ≥ 0.05%), occurring in 199 samples (13.1% 199/1518). Comparative analysis between stable (*n* = 1,319) and unstable (*n* = 199) groups demonstrated significant differences (*p* < 0.05) in most variables except the concentration of Calcium, Magnesium, Lipid, Lipid composition and Trace elements. Complete statistical comparisons are detailed in Table 2.

**Table 2:** The characteristics of variables in stability and instability group.

Continuous variables	Stability (*n* = 1,319)	Instability (*n* = 199)	*p*-value
	Mean ± SD		
Temperature (°C)	15.9 ± 11.8	19.0 ± 11.9	0.002
Times (h)	82.1 ± 80.0	62.3 ± 76.3	0.002
Potassium (mmol/L)	37.2 ± 33.4	21.1 ± 24.0	0.000
Sodium (mmol/L)	68.5 ± 61.2	102.7 ± 82.4	0.000
Calcium (mmol/L)	12.3 ± 12.0	12.9 ± 11.0	0.848
Magnesium (mmol/L)	5.4 ± 5.4	4.9 ± 3.6	0.242
Phosphate (mmol/L)	13.8 ± 12.3	8.8 ± 8.6	0.000
Chloride (mmol/L)	50.6 ± 74.7	101.1 ± 91.5	0.000
Amino (g/dl)	3.6 ± 2.1	2.7 ± 1.2	0.000
Dextrose (g/dl)	11.5 ± 6.2	8.8 ± 4.9	0.000
Lipid (g/dl)	2.8 ± 1.0	2.7 ± 1.0	0.245
CAN	1237.8 ± 895.6	1261.1 ± 704.5	0.018

Categorical variables		*n* (%)	*n* (%)	*p*-value
Lipid composition	LCT 20%	269 (20.4)	35 (17.6)	0.117
	MCT/LCT 20%	923 (70.0)	164 (82.4)	
	SMOFlipid 20%	127 (9.6)	0 (0.0)	
Fat-soluble vitamin	Without	724 (54.9)	81 (40.7)	0.000
	Within	595 (45.1)	118 (59.3)	
Water-soluble vitamins	Without	676 (51.3)	81 (40.7)	0.007
	Within	643 (48.7)	118 (59.3)	
Trace elements	Without	719 (54.5)	99 (49.7)	0.238
	Within	600 (45.5)	100 (50.3)	
Heparin	Without	1,144 (86.7)	153 (76.9)	0.000
	Within	175 (13.3)	46 (23.1)	

### Machine learning prediction of PN lipid stability

**Figure 2:**
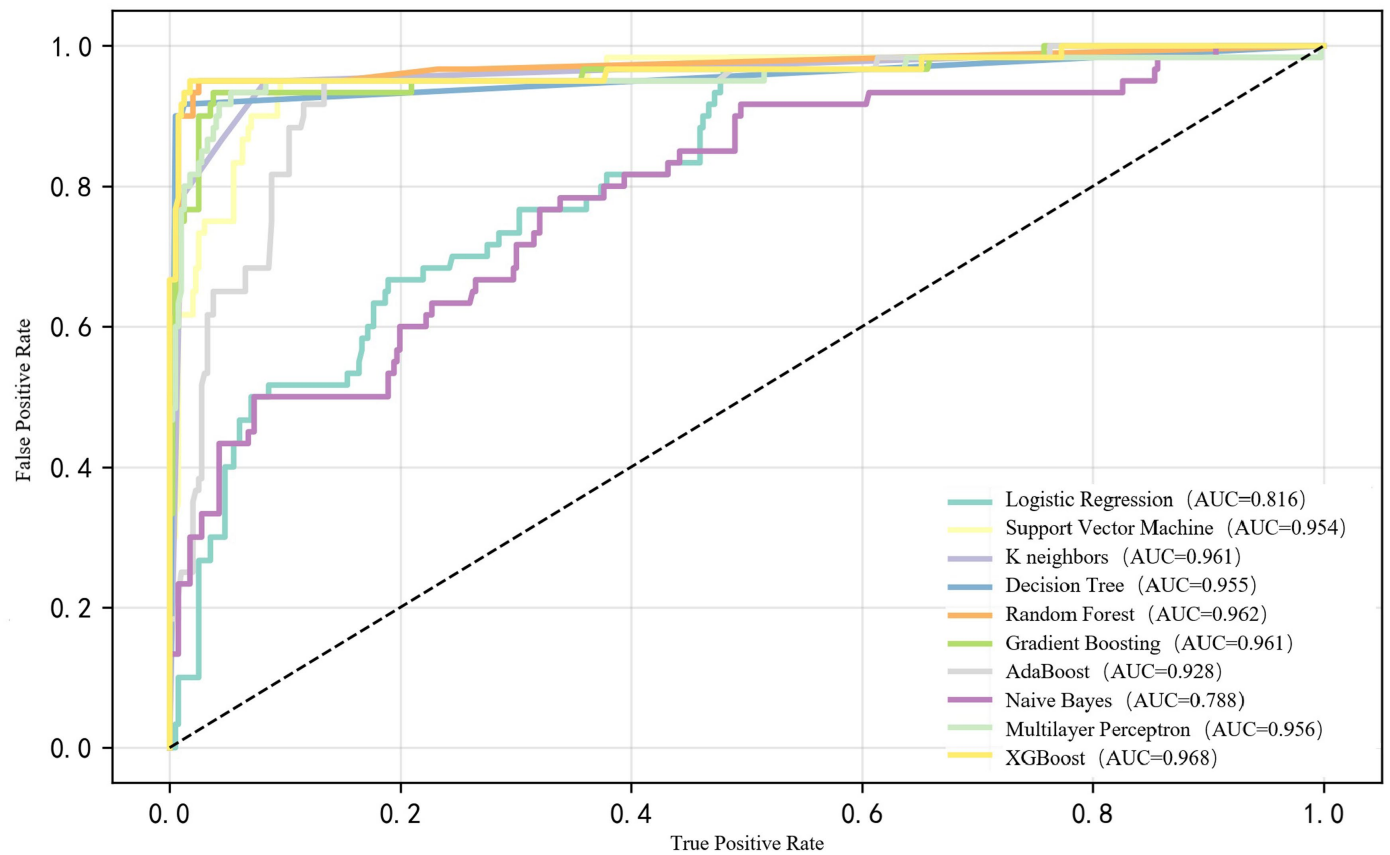
The ROC curve comparison of ten prediction models.

We compared the performance of ML models to predict PN lipid stability using ROC analysis. The AUC-ROC of the XGBoost model had the highest predictive value (AUC-ROC: 0.968, Accuracy: 0.980), followed by a random forest model (AUC-ROC: 0.962) and Gradient Boosting (AUC-ROC: 0.961). Detailed performance measures for all models are provided in Supplementary Table S1. We demonstrated the ROC curve comparison of 10 prediction models (Figure 2). Imbalance treatment optimization (Supplementary Table S2) confirmed SMOTE significantly enhanced XGBoost performance versus alternative methods. Finally, the XGBoost model achieved a recall of 0.98, a Precision of 0.98 and F1 score of 0.98.

Detailed mechanism of XGBoost: Algorithm Principle, An ensemble learning method based on gradient-boosted decision trees, which builds a strong classifier by sequentially training multiple weak learners; Key Parameters, max_depth = 6 (controls tree depth to balance bias and variance), learning_rate = 0.1, n_estimators = 100 (number of trees); Objective Function, Obj = *Σ* l (yᵢ, ŷᵢ) + Σ *Ω*(ƒₖ), where “l” is the loss function and “Ω” is the regularization term; Feature Interaction, Capable of automatically learning non-linear interaction relationships between features without manual feature engineering.

The test set confusion matrix shows: True Positives (correctly identified unstable cases) = 55, False Negatives (unstable cases misclassified as stable) = 4. True Negatives (correctly identified stable cases) = 392, False Positives (stable cases misclassified as unstable) = 5. This results in a Sensitivity/Recall of 93.2% (55/59) and a Specificity of 98.7% (392/397). We demonstrated the confusion matrix of SMOTE+XGBoost model (Supplementary Figure S2).

### Feature importance analysis

**Figure 3:**
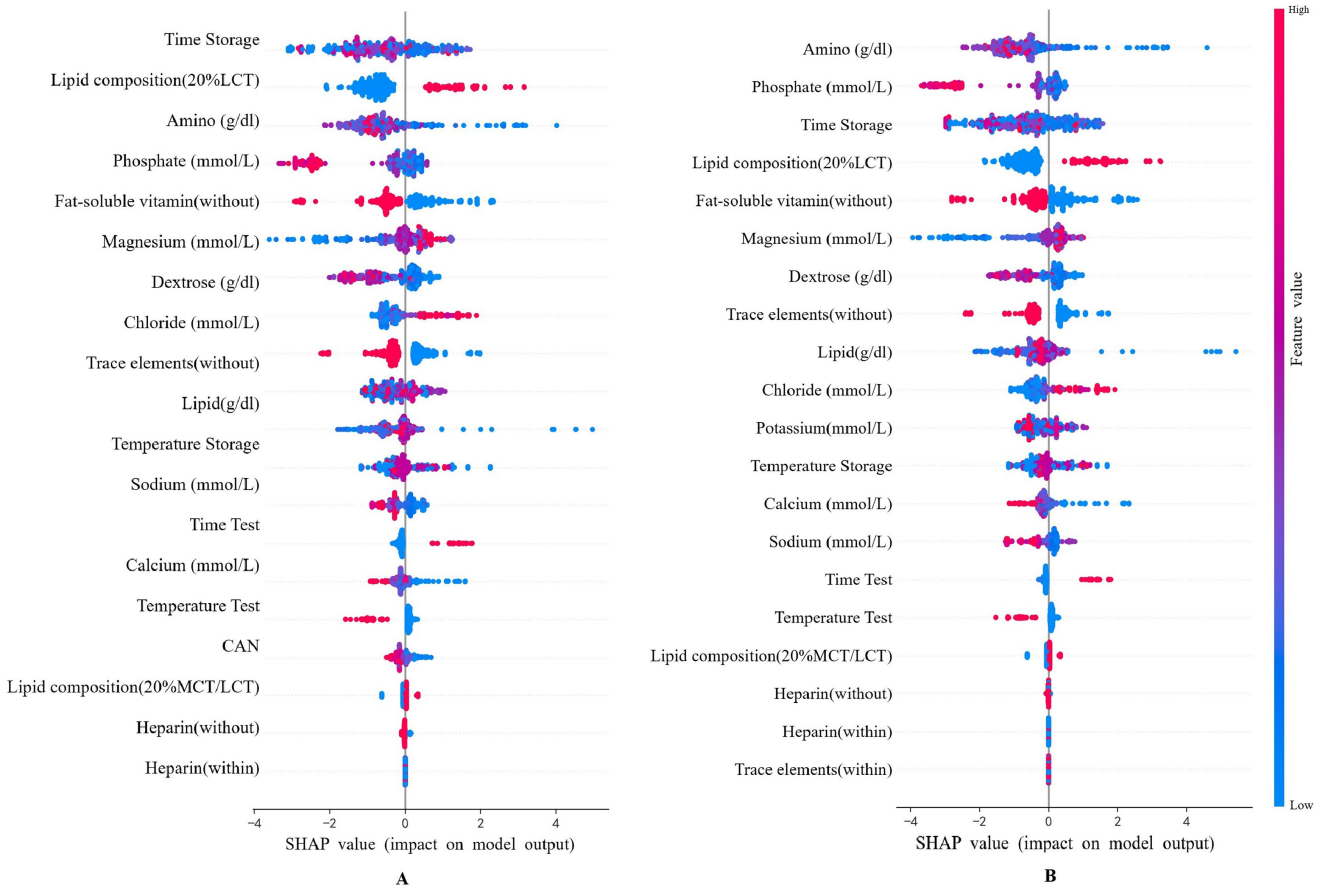
Features SHAP analysis in the XGBoost model. **(A)** No handling of multicollinearity; **(B)** Deal with multicollinearity.

To provide recommendations for practice with a straightforward understanding of key features related to predicting lipid stability, SHAP analysis of the optimal XGBoost model identified key determinants of lipid emulsion stability with or without Multicollinearity handing (Figure 3). Amino, Phosphate, Dextrose, Time storage and Lipid composition demonstrated predominant predictive influence.

## Discussion

### Core findings and model innovation

This study established an XGBoost-driven multimodal data fusion framework to achieve high-accuracy prediction of lipid emulsion physical stability in PN. The model demonstrated exceptional performance on the test set, significantly outperforming conventional logistic regression and random forest. Key innovations include intelligent multisource data integration through transfer learning and domain adaptation, resolving heterogeneity from environmental conditions and prescription differences by enabling equivalent data transformation across 19 studies. Optimized Feature Engineering, 17 stability relevant features were extracted.

To address the potential impact of multicollinearity among input variables (as shown in Supplementary Figure S1), we systematically evaluated and managed feature correlations during data preprocessing. Variables were carefully reviewed, and those with lower physicochemical relevance (e.g., CAN) were excluded. A comparative sensitivity analysis of SHAP values was conducted before and after this multicollinearity handling. Although the ranking of specific features saw minor shifts, the overall importance trend remained largely consistent. This process refined the contribution weight of individual variables to the model, thereby enhancing the robustness and interpretability of our final model.

Although multiple continuous variables showed statistically significant differences between the stable and unstable PN groups (Table 2), their inherent variability and intercorrelations make it difficult to determine the stability of lipid in individualized TNA. This further highlights the advantage of using a ML approach, which can integrate multidimensional interactions among features—such as amino acids, dextrose, and electrolytes—to produce a robust predictive model. Importantly, the SHAP analysis reaffirmed the model’s ability to synthesize these interrelated influences, with key factors (e.g., amino acids, dextrose, and storage time) consistently emerging as major contributors, thereby validating the model’s interpretability and clinical relevance.

The performance gain achieved through the SMOTE-based resampling strategy can be attributed to the intrinsic characteristics of our dataset. SMOTE-generated synthetic samples effectively populate sparse regions within the feature space. Consequently, SMOTE’s interpolation mechanism effectively populated these sparse regions with plausible synthetic samples, which significantly enhanced the model’s ability to identify the minority class. This approach elevated the AUC from 0.95 to 0.97 and elevated the Accuracy from 0.97 to 0.98, thereby ensuring the predictive safety of the model. We add a sensitivity analysis regarding key SMOTE parameters in Supplementary Table S3.

### Analysis of key features and mechanisms

The stability of lipid in TNA is most significantly influenced by the concentration of amino acids, which play a crucial role in regulating the pH of TNA. This study first identifies phosphate concentration as a novel predictor of PN lipid stability through ML modeling—a previously unreported association whose mechanism warrants further investigation, though we hypothesize involvement of phosphate groups in the emulsion’s hydrophilic layer. Following features such as concentration of others electrolyte and dextrose, type of lipid, CAN, or storage time that have been largely evaluated and confirmed as relevant predictors of lipid stability in PN. CAN was excluded due to multicollinearity handling. This was also discovered in all models SHAP analysis (Supplementary Figure S3). Cations can disrupt the electrostatic repulsion between lipid droplets, thereby compromising the stability of lipid emulsions, though modulated by compositional factors (6). The 100-fold higher aqueous solubility of medium-chain caprylic acid (C_8_) versus palmitic acid (C_16_) (7), reduces oil-water interfacial tension, explaining the low stability of LCT emulsions (8, 9) observed in our model. While temperature and storage duration influence stability, their limited predictive role may relate to anomalous PFAT5 reduction over time—potentially attributable to compounding-induced air bubbles (10). Our stability assessment aligned with USP and Chinese Pharmacopoeia standards using MDD/PFAT5, as enlarged droplets (>5 μm) pose embolism risks: murine studies demonstrate PFAT5 > 0.05% causes microvascular damage and reticuloendothelial injury (11), consistent with clinical reports of pulmonary fat deposition in neonates (12); critically, PFAT5 > 0.4% induces phase separation visible as surface oil droplets (1), representing immediate life-threatening risks.

### Value in clinical practice

As shown above, there has a critical impact of PN stability on patient safety, particularly the complex physicochemical behavior of lipid emulsions influenced by multifactorial interactions. In clinical practice, there are complex decisions in medical order, prescription review, PN preparation and PN safety monitoring. This ML-driven solution empowers pharmacists and physicians to rapidly evaluate PN compatibility by analyzing the influencing factors of specific prescriptions. ML has been increasingly used in the field of nutrition science, considering the diversity and complexity of individuals, ML can be used to predict precision nutrition support through multivariate and high-dimensional data analysis. In 2022–2027, the National Institutes of Health of the United States will provide $170 million to fund the development of algorithms for precision nutrition support (13). Our work provides a distinct contribution. Rather than merely applying XGBoost model, this study establishes a comprehensive, ML-enabled meta-analysis framework tailored specifically to PN stability. Our approach integrates a uniquely standardized clinical dataset, domain-specific feature engineering that combines environmental, electrolyte, and macronutrient variables, and an interpretable predictive tool with direct clinical utility. By adapting ML techniques to this specific challenge, we offer not only a practical system for stability prediction but also a reusable methodological framework for studying complex systems stability in pharmaceutical and nutrition sciences.

### Limitations and strengths

As a retrospective investigation, this study necessitates further validation through prospective multi-laboratory trials, particularly to verify phosphate’s mechanistic role in emulsion destabilization. In addition, lipid emulsion stability parameters were insufficient: pH, Zeta potential, peroxide parameter, and free fatty acid were lacking, but we followed scientific physical stability verification criteria. Subsequently, extended multi-task learning was used to predict calcium and phosphorus precipitation, vitamin degradation and other stability risks.

ML approach overcomes the clinical impracticality of experimental verification for individualized PN formulations by modeling multifactorial interactions across heterogeneous datasets. Future research will expand training data to enhance generalizability and integrate physical–chemical stability predictions, ultimately constructing a comprehensive risk assessment paradigm for PN quality assurance.

## Conclusion

This study establishes an XGBoost-driven multimodal fusion framework that transforms opaque model predictions into clinically actionable insights via SHAP interpretability. By resolving cross-laboratory data integration barriers in predicting lipid emulsion stability, we deliver a high-accuracy, explainable decision tool for clinical practice. The synergistic effect of key role of Phosphate, Dextrose, Sodium and Lipid composition (MCT/LCT) provide evidence-based reference for individualized PN formulation review. This study delivers both a high-accuracy predictive model for lipid emulsion stability in PN and a pioneering research strategy for assessing stability in complex pharmaceutical formulations.

## Data Availability

The raw data supporting the conclusions of this article will be made available by the authors, without undue reservation.
