# Supplementary material for: Machine learning predicts lipid emulsion stability in parenteral nutrition using multi-laboratory literature data
Source: Front Nutr. 2025 Nov 17;12:1668464. doi: 10.3389/fnut.2025.1668464 (PMC12667437; doi:10.3389/fnut.2025.1668464)
Supplement: Supplementary file 1 [file Table_1.DOCX]

Table S1. Model performance comparison (in order of accuracy)

|  | **Models** | **Accuracy** | **Precision** | **Recall** | **F1** | **AUC-ROC** |
| --- | --- | --- | --- | --- | --- | --- |
| 1 | XGBoost | 0.982 | 0.980 | 0.980 | 0.982 | 0.968 |
| 2 | Decision Tree | 0.978 | 0.978 | 0.978 | 0.978 | 0.955 |
| 3 | Random Forest | 0.978 | 0.978 | 0.978 | 0.978 | 0.962 |
| 4 | Gradient Boosting | 0.952 | 0.959 | 0.952 | 0.954 | 0.961 |
| 5 | Multilayer Perceptron | 0.952 | 0.956 | 0.952 | 0.953 | 0.956 |
| 6 | KN neighbors | 0.930 | 0.945 | 0.930 | 0.934 | 0.961 |
| 7 | Support Vector Machine | 0.886 | 0.932 | 0.886 | 0.898 | 0.954 |
| 8 | AdaBoost | 0.886 | 0.923 | 0.886 | 0.897 | 0.928 |
| 9 | Logistic Regression | 0.711 | 0.858 | 0.711 | 0.755 | 0.816 |
| 10 | Naive Bayes | 0.353 | 0.858 | 0.353 | 0.397 | 0.788 |

Table S2. Effects of different imbalance treatment methods on the XGBoost model

| **methods** | **Accuracy** | **Precision** | **Recall** | **F1** | **AUC-ROC** |
| --- | --- | --- | --- | --- | --- |
| Raw data | 0.972 | 0.972 | 0.972 | 0.972 | 0.954 |
| SMOTE | 0.982 | 0.980 | 0.980 | 0.982 | 0.968 |
| ADASYN | 0.980 | 0.980 | 0.980 | 0.980 | 0.966 |
| Random Under sampling | 0.917 | 0.941 | 0.917 | 0.923 | 0.960 |

Table S3. The sensitivity analysis regarding key SMOTE parameters

| **k_neighbors** | **sampling_strategy** | **Accuracy** | **F1** | **AUC-ROC** |
| --- | --- | --- | --- | --- |
| 3 | auto | 0.982 | 0.982 | 0.966 |
| 3 | 0.5 | 0.982 | 0.982 | 0.968 |
| 3 | 0.8 | 0.982 | 0.982 | 0.967 |
| 3 | 1 | 0.982 | 0.982 | 0.966 |
| 5 | auto | 0.980 | 0.980 | 0.968 |
| 5 | 0.5 | 0.978 | 0.978 | 0.967 |
| 5 | 0.8 | 0.982 | 0.982 | 0.966 |
| 5 | 1 | 0.980 | 0.980 | 0.968 |
| 7 | auto | 0.982 | 0.982 | 0.967 |
| 7 | 0.5 | 0.980 | 0.980 | 0.967 |
| 7 | 0.8 | 0.980 | 0.980 | 0.967 |
| 7 | 1 | 0.982 | 0.982 | 0.967 |
| 11 | auto | 0.976 | 0.976 | 0.969 |
| 11 | 0.5 | 0.978 | 0.978 | 0.965 |
| 11 | 0.8 | 0.980 | 0.980 | 0.965 |
| 11 | 1 | 0.976 | 0.976 | 0.969 |


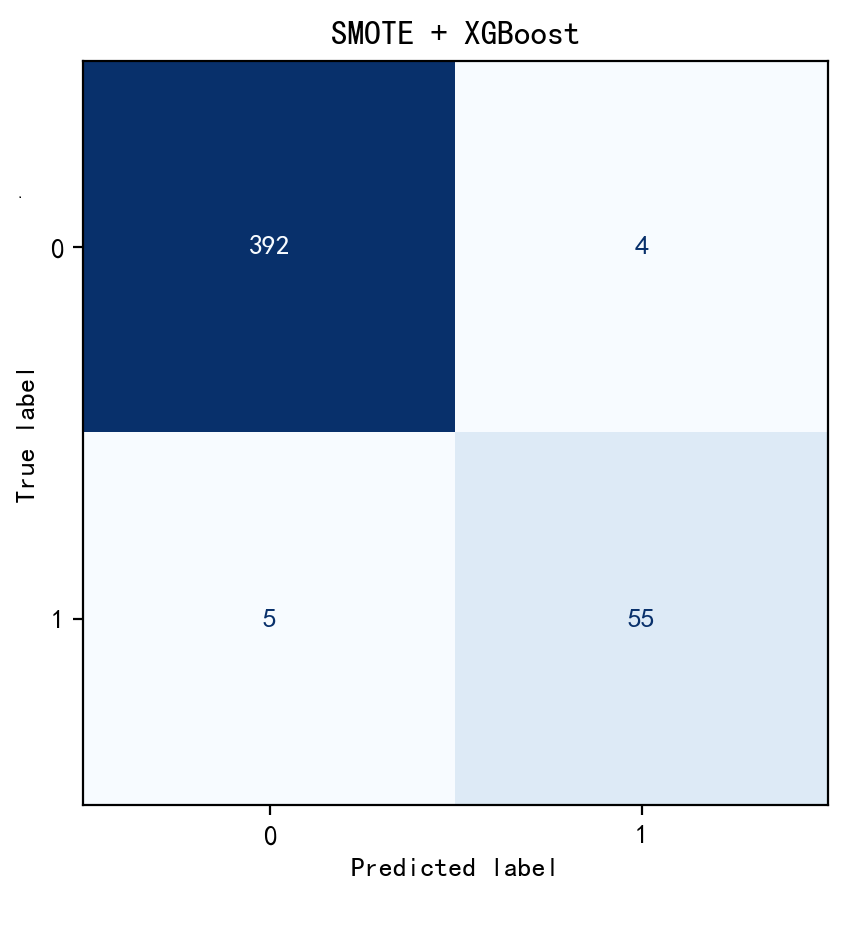

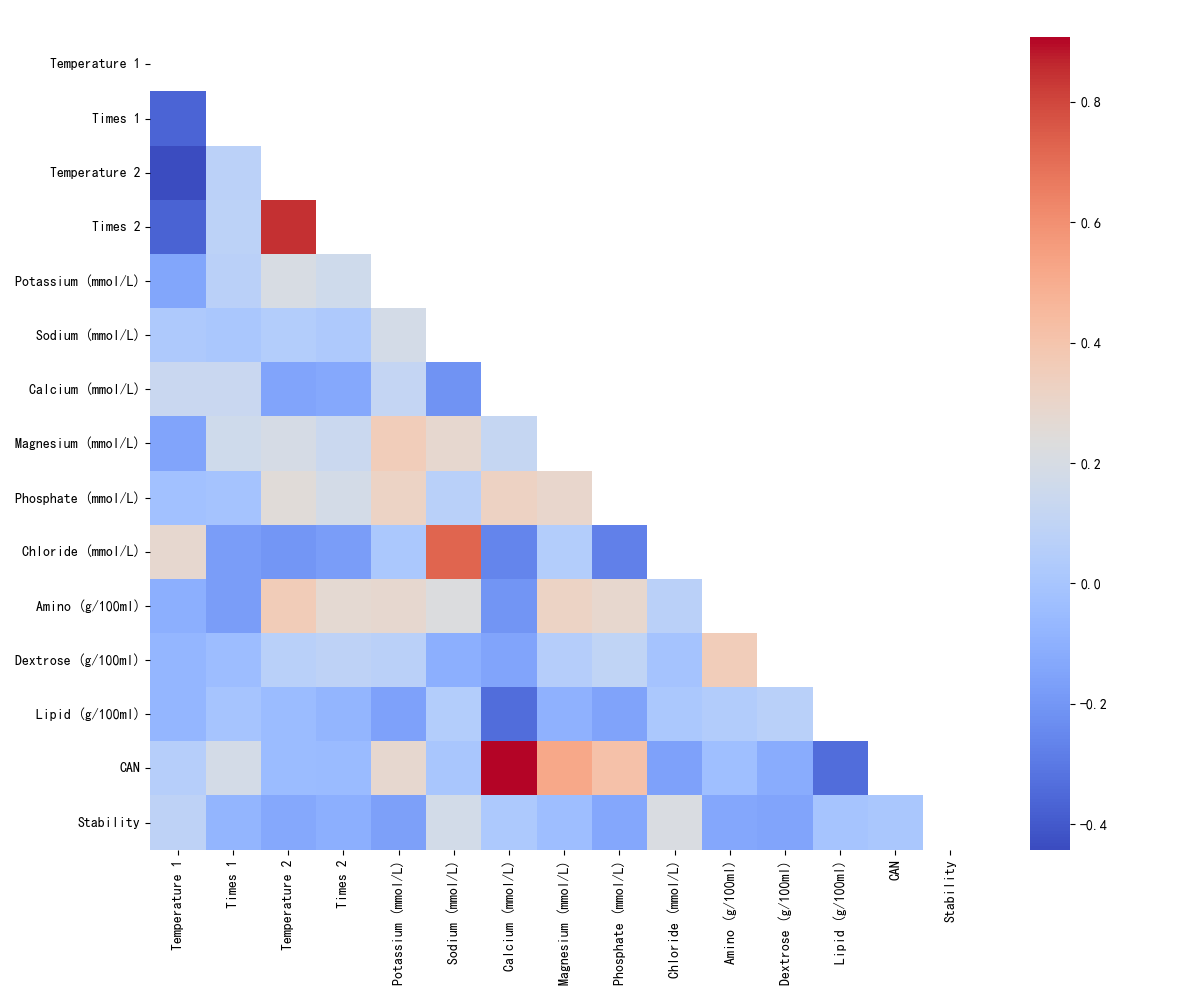
Figure S1. The correlation heatmap of features extracted from the experimental studies

Figure S2. The confusion matrix of SMOTE+XGBoost model


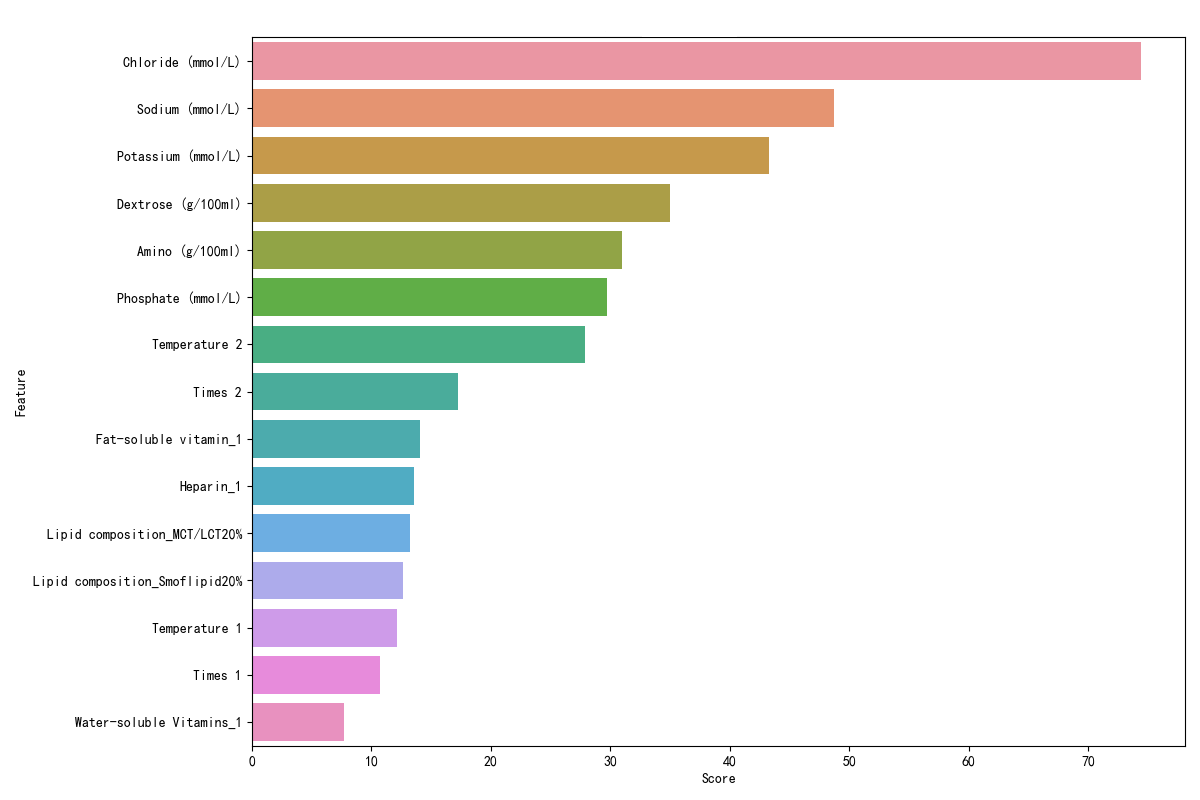


Figure S3. Features SHAP analysis in all models
